# Understanding system barriers and facilitators in transnational clinical cancer research: The value of rapid and multimodal ethnographic inquiry

**DOI:** 10.3389/fsoc.2022.991183

**Published:** 2022-12-01

**Authors:** Kristin Bright

**Affiliations:** ^1^Department of Anthropology, Middlebury College, Middlebury, VT, United States; ^2^Department of Anthropology, University of Toronto, Toronto, ON, Canada

**Keywords:** rapid ethnographic research, multimodal ethnography, clinical trial, breast cancer, transnational, treatment delay, system delays, system change

## Abstract

**Introduction:**

In middle and low resource countries worldwide, up to 70% of breast cancer cases are diagnosed as locally advanced (stages IIB-IIIC). Delays in referral from primary to specialty care have been shown to prolong routes to diagnosis and may be associated with higher burdens of advanced disease, but specific clinical and organizational barriers are not well understood.

**Methods:**

This article reports on the use of rapid ethnographic research (RER) within a largescale clinical trial for locally advanced breast cancer (LABC) in India, Mexico, South Africa, and the US. Our purpose is twofold. First, we demonstrate the value of ethnography as a mode of *evaluative listening*: appraising the perspectives of diverse patients and clinicians regarding prolonged routes to LABC diagnosis and treatment. Second, we show the value of ethnography as a *compass for navigating* among discrepant clinical research styles, IRB protocols, and institutional norms and practices. We discuss advantages and limits involved in each use of RER.

**Results:**

On the one hand, ethnographic interviews carried out before and during the clinical trial enabled more regular communication among investigators and research sites. On the other hand, the logistics of doing the trial placed limits on the extent and duration of inductive, immersive inquiry characteristic of traditional fieldwork. As a partial solution to this problem, we developed a *multimodal ethnographic research* (MER) approach, an augmentation of video-chat, phone, text, and email carried out with, and built upon the initial connections established in, the in-person fieldwork. This style has its limits; but it did allow us to materially improve the ways in which the medical research proceeded.

**Discussion:**

In conclusion, we highlight the value of not deferring to a presumed incommensurability of ethnographic fieldwork and clinical trialwork while still being appropriately responsive to moments when the two approaches should be kept apart.

## Introduction

Breast cancer is the fifth-leading cause of death worldwide, resulting in 685,000 deaths per year (American Cancer Society (ACS), 2022; World Health Organization (WHO), 2022). Up to 70% of breast cancer cases in middle and lower-resource countries are locally advanced (stages IIB-IIIC, or invasive disease with regional spread) (Unger-Saldaña, 2014; Balogun and Formenti, 2015). In Mexico, breast cancer is one of the main causes of death in younger women (Villarreal-Garza et al., 2019) and women under 40 years of age are significantly more likely than post-menopausal women to be diagnosed with advanced stage disease and triple-negative disease (a more aggressive tumor that is harder to treat) (Villarreal-Garza et al., 2017). Despite 50% of individuals in Mexico having access to national health insurance, women with a suspected case of breast disease face significant clinical barriers (Bright et al., 2011). While individuals may postpone consulting a physician for a number of reasons including concerns about cost, mistrust of medical providers, lack of childcare, or concerns about missing work or being fired, healthcare system issues including referral delays or multiple extraneous appointments between first presentation and initiation of treatment are associated with prolonged routes to diagnosis (Bright et al., 2011; Unger-Saldaña et al., 2019) and may be associated with advanced stage disease and disease progression (Caplan, 2014). In the United States, Black, Indigenous, Latinx, Asian American, and Pacific Islander women with breast cancer are more likely to be diagnosed with advanced disease and experience a higher rate of cancer death at younger ages than white women (Hendrick et al., 2021). Despite clinical advances over the past 20 years, Black women are still two times more likely to die from breast cancer than white women (American Society of Clinical Oncology (ASCO, 2022). Cancer epidemiologists urge more investment in national and subnational cancer resources but are not well equipped to uncover the complex systems and interactions that underlie cancer (e.g., biological, behavioral, social, economic) (Mabry et al., 2022). Beneath epidemiological recommendations is a complex patchwork of political and institutional personalities, goals, and assumed ways of doing things, many of which are so tightly woven into medical life as to seem invisible or insignificant.

Ethnography is a critical tool for teasing apart the complex meanings and structures of power that inform cancer research and treatment (Petryna, 2009; Joseph and Dohan, 2012; Livingston, 2012; Burke, 2014; Bright, 2015; Caduff et al., 2018; Banerjee, 2020). However, traditional fieldwork depends on months or years of immersive observation, interviewing, fieldnoting, and thick description to characterize complex layers of lived experience and historical context. Such methods take time and money and typically rely on one investigator. By contrast, approaches such as rapid ethnographic research (RER) are usually team-based and take place in intensive bursts of several weeks or months. The potential speed, recursivity, and collaborative style of these approaches make them useful in research where multiple agendas and investigators are involved. Moreover, the quick turnaround of findings is appealing in clinical and public health settings where timely results can lead to positive change in patient health and institutional success (Vindrola-Padros and Vindrola-Padros, 2018; Palinkas et al., 2020; Sangaramoorthy and Kroeger, 2020).

In this article, we report on the rapid ethnographic research we carried out as part of a large, transnational study of locally advanced breast cancer in India, Mexico, South Africa, and the US. The original aim of the study was to build a biological and clinical description of locally advanced breast cancer (LABC) in a multinational, multiethnic cohort and to do so in tandem with a phase I/II trial to assess clinical response to concurrent chemotherapy (paclitaxel) and radiation followed by surgery (Formenti et al., 2003). Over the course of the collaboration, more than 50 investigators from fields of anthropology, biology, biostatistics, epidemiology, medical oncology, pathology, radiation oncology, and surgical oncology participated across five centers in four countries (India, Mexico, South Africa, and the US) (see also Connolly et al., 2006; Braunstein et al., 2007; Adams et al., 2010; Arslan et al., 2012 for clinical and molecular outcomes associated with the trial including the identification of unique alterations in protein synthesis underlying the development of LABC).

The idea to carry out rapid ethnographic research (RER) as part of the broader international project emerged early on and was strongly supported by our clinical and molecular science colleagues. In light of the high burden of LABC in middle and low resource settings, investigators agreed it was ethically imperative to ask how clinical and organizational factors contribute to prolonged routes to diagnosis and what can be done to mitigate those. From an anthropological standpoint, we were keen to understand the relationship between explanations of illness and structural factors such as access to primary care, insurance, employment, childcare, transportation, wellness, and safety. In regard to the logistics of the clinical trial, we were interested to understand more effective and equitable routes to LABC presentation, clinical research access, and specialized treatment.

To paint a picture of this tandem project (ethnographic fieldwork and clinical trialwork), we first examine the design of the RER and its practical and epistemological goals. We then discuss some of our key findings including the types of institutional barriers most concerning for access to care and potential strategies for change. On the one hand, ethnographic interviews carried out in the weeks before and during the trial in each site (India, Mexico, South Africa, US) created a regular and confidential space for discussion of differences in research style, ethics, institutional practices, and resource needs. On the other hand, the logistics of doing the trial placed limits on the duration and extent of immersive inquiry characteristic of traditional fieldwork. Furthermore, not all of our ethnographic data were favorable to the goals of the clinical study or its continuation across sites, even as our findings showed that (most) barriers could be navigated. To our fast-paced clinical colleagues, structural barriers often seemed bewildering and something to be circumvented. But as research made clear, the differences simmering beneath the surface of the trial could be as much a site for the discovery of workable solutions, as a place to move on from. In the sections below, we examine the benefits of RER for cross-disciplinary co-learning and nimbleness; that is, the value of not deferring to a presumed incommensurability of fieldwork and trialwork while still being appropriately responsive to moments when those two roles or approaches should be kept apart.

## Methods

### Setting the scene

#### The clinical trial design and procedures

Our rapid ethnographic research (RER) took place within a transnational, multi-investigator study of locally advanced breast cancer based at the New York University School of Medicine in 2005 to 2018 and carried out at clinical centers in India, Mexico, South Africa, and the US. The study was funded by the US Congressionally Directed Medical Research Program and aimed to understand the progression of breast cancer from local disease to metastasis by asking whether LABC that responds to a specific, uniform therapy is genetically, immunologically, and molecularly distinct from that which is unresponsive and progresses to metastatic disease. With the understanding that LABC is a multidimensional global disease that disproportionately impacts minoritized communities and lower-income patients, the study was one of the first to investigate LABC in an internationally diverse cohort using a multidisciplinary clinical, biological, sociocultural, and health systems approach.

The clinical study, specifically, was a phase I/II trial to assess clinical response to concurrent chemotherapy (paclitaxel) and radiation followed by surgery (Formenti et al., 2003; Adams et al., 2010). Patient entry criteria consisted of patients over 18 years of age diagnosed with LABC (stages IIB-IIIC). Tumor staging was assessed by physical exam, mammography, and/or ultrasound; and all patients underwent further staging *via* computed tomographic (CT) scan and bone scan to exclude distant metastases. Eligible patients were invited to participate in the trial *via* informed consent obtained in adherence with each center's IRB and the local language(s) of each center (see discussion of IRBs below). Therapy consisted of 30 mg/m^2^ paclitaxel administered as a 1-h intravenous infusion twice weekly for 10–12 weeks, with external-beam radiation therapy initiated within 1 week of the first paclitaxel dose and delivered daily to the breast, axillary, and supraclavicular lymph nodes during weeks 2–7, at 1.8 Gy per fraction to a total dose of 45 Gy followed by a boost of 14 Gy at 2 Gy per fraction to the originally palpable tumor (Formenti et al., 2003; Adams et al., 2010). After FDA approval of trastuzumab in 2006, patients with HER-2 positive tumors received weekly trastuzumab (2 mg/kg) during paclitaxel treatment (Adams et al., 2010). Across the four study sites, 195 patients were screened for trial, 71 were enrolled to study, and 68 completed the clinical treatment protocol. This number was significantly lower than the original study participant target of 300. In the results section, we present institutional and organizational factors potentially shaping these lower rates of trial screening and enrollment and then discuss how those contribute to a wider rationale for RER companion studies to clinical cancer trials.

#### The ethnographic companion study

Prior to the launch of the clinical study (above), we crafted an ethnographic question capacious enough to appeal to diverse disciplinary interests in the broader study: “Given the high burden of advanced stage breast cancer diagnoses in clinically underserved settings, what is the role of institutional and organizational factors in delayed care for LABC?” To this, we added a second question about system change: “What if we could build into this international clinical collaboration a study of local system features, including ethical, existential, logistical, and public health dimensions that affect how people with breast cancer experience access to diagnosis, treatment, and post-treatment care?” We created a mixed-method ethnographic research design comprised of qualitative questions and quantitative measures. In addition to interval measurements of the time between when a person or physician first noticed a symptom to when a person started treatment, our qualitative aim was to understand diverse patient and provider experiences with symptom detection, follow up care, referrals, and other pathways to confirmed diagnosis. Integral to this, we sought to understand the specific barriers providers and patients confront when setting up, administering, or enrolling into an international clinical trial. This article focuses on these qualitative questions and does not report on the quantitative interval study which we report elsewhere (Bright et al., 2011).

While we sought to keep our ethnographic aims aligned with the wider group of 50 colleagues on the clinical and molecular study, we did not want to compromise the inductive science of ethnography or its potential for making underappreciated truths or information visible. Our intent was not to verify one epistemology against another (ethnographic inquiry vs. clinical response) or to set up a framework for external validity. Rather, our rationale for ethnographic research was its potential contribution to (1) the description of LABC transnationally, (2) the characterization of system factors impacting LABC experiences differently across sites, (3) the measure of prolonged routes to treatment, and (4) quicker translation of clinical research to public health.

### Ethnographic procedures and methods

We carried out ethnographic research prior to and during the launch of the clinical trial in each of the four study countries. Our university had generous support for medical students to gain experience in international health research, so we worked closely with 12 NYU medical students during the startup of the trial in each location. Because it was the students' first experience in ethnographic research, we organized a rapid four-week course with foci on global cancer inequalities and LABC, human subjects and ethics, mixed method ethnography, cultural awareness, and community engaged research. Following this training, the students and author spent an average of 6 weeks in each site (India, Mexico, South Africa) dividing our time into 3 weeks pre-trial and 3 weeks post-launch. In New York, we spent on average 3 weeks pre-trial and multiple months post-trial at two sites.

Our fieldwork methodology included participant observation, semi-structured interviews with providers and patients, and review of patient charts and registries. In total, the in-person RER portion included semi-structured interviews with 112 patients (India 32; Mexico 30; South Africa 16; New York 34) and semi-structured interviews with 42 providers (India: 9; Mexico: 10; South Africa: 6; New York: 17) including nurses, social workers, primary care physicians, surgical oncologists, medical oncologists, radiation oncologists, epidemiologists, and pathologists. Ethnographic observation including participation in ward rounds, tumor board meetings, surgeries, labs, and other meetings (average of 30 h pre- and 30 h post-trial for 240 h on average total). Each RER experience was participated in by one to three students from NYU, and two to three students from the local site, who assisted with chart review, data entry, and transcription.

Observations and interviews with providers were conducted in English (all sites other than Mexico) and Spanish (Mexico). Patient interviews were conducted in English, Spanish, Afrikaans, Malayalam, Chinese, Haitian Creole, and Russian. In-clinic translators and medical students at each center assisted in the collection of interviews and observations in languages other than English.

### Ethics and analysis

IRB approvals were obtained from participating centers Tygerberg Hospital (South Africa), Amrita Institute of Medical Sciences (India), Instituto Mexicano del Seguro Social (Mexico), Bellevue Hospital (US), and NYU Cancer Center (US), as well as from our study sponsor CDMRP-DOD. Verbal consent was obtained prior to interviews in the preferred language of the participant. Interviews lasted 30 to 45 min and were audio recorded with participant consent. Patient and provider interviews explored perceptions of and experiences with symptom discovery, symptom explanation, efforts to seek care, barriers to care, confirmation of diagnosis, and expectations about treatment. In the absence of an official tumor registry in two of the five sites, we conducted one-year retrospective chart review of new breast cancer cases to understand the proportion of LABC to earlier stage cancers. Data retrieved from charts included tumor staging, disease-related information, and time intervals between symptom detection, initial visit, diagnosis, and start of treatment. Interval data were entered into excel. Descriptive data were recorded in notebooks, word, and excel.

We carried out partial manual transcription of interview recordings and observational data and then reviewed the transcripts and notes to develop a coding schema based on salient themes including perceived barriers to diagnosis and treatment and local institutional issues impeding access to care or continuity of care. We then used social network analysis to assess structural barriers and potential leverage for system change (Rapport et al., 2020). Given the considerable variation in clinical structures at each site, and in healthcare systems in each country, we aimed to identify idiosyncratic system features in LABC diagnosis or treatment that would be particularly important to examine more closely before system improvements could be made.

### The shift to multimodal ethnographic research

After the initial in-person work was completed, we shifted to digital modes of communication and information gathering including video chat, phone, text, and email. We met twice monthly *via* conference calls with clinicians and research coordinators in the sites abroad. In New York, we continued to meet weekly with our clinical and scientific colleagues. Friday morning meetings of our multidisciplinary breast research group provided opportunity to exchange notes with colleagues and observe system barriers. While in-person fieldwork lasted 6 weeks on average in each site, our use of multimodal ethnographic research (MER) including digital technologies for informal interviewing, needs assessment, and organizational study enabled us to continue adding to and deepening our analysis of system barriers up until the study closed in 2018.

## Results and findings

### Communication barriers

Overall, our findings revealed a disturbing pattern of healthcare system barriers that we have reported previously (Bright et al., 2008, 2011; Yip et al., 2011; Bright, 2015). The most common barriers identified by patients and providers were insufficient training at the primary care level for evaluation of symptoms or referral to care, mismanagement of suspicious lumps, underutilized pathology, and delay in referral to imaging and/or diagnostic exam. In addition, patients described political economic barriers including lack of health services in their neighborhood or township, lack of transportation, too costly transportation, job precarity, lack of insurance or money for care, geographic distance from home to clinic, and gender inequality (e.g., in South Africa where several participants were uncertain whether they could seek care or start treatment due to insufficient support from employers, husbands, and/or tribal leaders). Findings diverged across national settings as well. While patterns in presentation to diagnosis appeared significant across all study sites in the US, patients in India, Mexico, and South Africa more frequently reported infrastructure barriers including lack of specialty care and coordination of care, prevailing societal perceptions about women's health as a low priority, lack of family savings for health care, and distance between patients' homes and cancer treatment centers (as far as 500 km).

In this section we focus on RER participants' difficulties with in-clinic communication about treatment and clinical trial screening. We had expected that friendly, linguistically appropriate navigation would help support patients with complex, sometimes scary procedures such as biopsy, surgery, and chemo-radiation—as well as reduce stress associated with the processing of large amounts of new information, promote interaction with the clinical research team, and enable patients to feel more prepared to make decisions about trial participation. In a prior study at our collaborating site in New York, Chinese American breast cancer survivors had reported multiple barriers during interactions with clinical staff and a majority reported unmet information needs (Eaton et al., 2017). Our RER findings in this study revealed that patients experienced similar gaps and that those occurred across three dimensions of communication: translational, structural, and decisional.

RER participants agreed that navigation in one's preferred language was vital, along with effective translation of clinical procedures in plain language. But RER participants also spoke about unmet needs in communication structure as well, including gaps in what clinicians understood or were curious to learn about patients' cultural and/or religious explanations about particular physical conditions; variations in social norms regarding authority, voice, turn-taking, and forthcomingness during clinical interactions; and patients' time and transportation limitations when it comes to multiple appointments. In settings like New York, where English-speaking physicians routinely interact with non-English speaking patients, additional problems cropped up when patients experienced too much or too little information exchange during clinical trial screening. Patients across the four sites reported that the experience of either “rushing through things” or “information overload” directly impacted their decision to decline or feel unsure about trial participation.

Beyond these two levels, a third level of communication was salient for RER participants: *decisional* communication. Among patients who had reported either very few *or* very diverse experiences with clinicians in the past—including for example multiple interactions with traditional healers, public health agencies, and/or primary clinics—there were additional barriers to navigate around decision making, an aspect of care that shifts in salience when treatment or a clinical trial is offered. Many patients explained that a yes or no answer was sufficient, for example, and did not perceive clinical trial screening interactions as a site where one could ask questions. There was also some confusion among clinicians regarding what patients consider to be a decision in the first place and how those various meanings and expectations would shape discussions about clinical trial options. RER patients and clinicians agreed that informed decision making needed to take place across all three registers of communication: translational, structural, and decisional. There was room, some participants added, for more open and creative uses of digital modalities, along with non-verbal demonstrations including whiteboard diagrams, surgical video demonstrations, e-health, and other tools.

### Institutional flashpoints

In addition to barriers at the healthcare system level and in-clinic communication issues, institutional research barriers were noted across the sites by RER participants. In the following sections, we focus on the experiences of the clinical trial investigators at the four locations. Particularly for the initial stages of the study, moments of conflict springing from epistemological differences in the aspirations of investigators, study sponsors, and hospital administrators played out differently depending on the organization of the relationships between these groups of stakeholders, including (but not limited to) their relative power. Because of the effect of these ignition points on the very possibility of collaboration, we examine their implications for global cancer and cancer healthcare system change in the discussion section below.

#### “How long is a piece of string?”: Negotiating the meaning of consent

In the early days of the collaboration, before the clinical trial, we sought approval from Institutional Review Boards (IRBs) at each site. Protocols were drawn up to ensure that informed and fully voluntary consent was gathered from eligible trial participants. Colleagues across the sites were united in their concern that the trial should fit as seamlessly as possible into existing hospital activities. The shared nature of these concerns did not mean unanimity in how to address them. In most cases, differences centered on the scope and content of ethics protocols and consent forms.

Located on the outskirts of Cape Town, Tygerberg Hospital is the second largest public specialty hospital in South Africa. With unemployment as high as 80% in some of the Western Cape, many people rely on Tygerberg Hospital for most or all of their health care. Less than 10% of people in Cape Town have health insurance or health savings accounts, and those who do tend to be wealthy, white, and with access to their own transportation. By contrast, patients at Tygerberg tend to come from underserved rural areas and townships. About 60% are mixed race, 15% Black, 15% white, and 10% East Indian. Nearly all speak Afrikaans and have limited access to cancer health information and social, financial, and transportation resources for screening and treatment seeking.

In the weeks prior to the LABC trial, we spoke on nearly a daily basis with the surgical oncologist leading the local study. On one occasion, we were having a conversation about IRB approval (one of many, as it turned out). We asked whether the process for our trial was different from the usual procedure at Tygerberg. “How long is a piece of string?” he grimaced, “of course it is different! The biggest thing is that it [the study sponsor CDMRP] requires two sides of review, local and their own, and that makes it difficult. Because there is not a lot of congruence between what the institution needs and what it wants.” We asked what he meant. “To me, a wholly new experience was to obtain a certificate for the ‘handling of hazardous substances.' This was something that no one had ever thought of. It's not that we throw all our hazardous substances into the river, but we handle it in an entirely different way. What the study sponsor wanted, there is no precedent here. It inspired a lot of head-scratching.”

We asked whether he could think of any other instances of incongruence. He immediately responded, “consent forms.” He described how one of the most off-putting aspects of the trial was the labor (on his part) to satisfy stringent local protocols made doubly difficult by the study sponsor's stringent protocols in the US. To demonstrate this point, he read a passage to me from the Standard Operating Procedures and Guidelines of his hospital pertaining to consent: “the language used in the consent form should be familiar to the local community and easily understandable; the form must be written in clear simple language aimed at a maximum Standard 6 [Grade 8] reading level.”

Several months prior, during local IRB's review, our colleague was dismayed to learn that approval had been withheld due to improper translation of the consent documents in English and Afrikaans. This meant another month of revision, resubmission, and review by the local IRB, to be followed by yet another review by the study sponsor in the US. He continued, “this language problem is a classic example of the IRB being difficult. There's someone on our IRB who has a language bee in their bonnet. They want to have the language correct, so it's understood correctly. But it's the level of scrutiny. What they're taking objection to is just too much. They're telling us where to put the punctuation, and they want us to follow language patterns from 100 years ago [more formal Afrikaans speech patterns].” Summarizing his experience, he said with a sigh, “it all just confirmed my prejudices regarding regulatory concerns. As usual, it was a pain in the behind, and it introduced a whole new level of harassment.”

This perception was echoed by a surgeon at the collaborating site in Cochin. He described a serious conflict in the consent process. On the one hand, most patients preferred to arrive at a decision about treatment (including participation in clinical research) through collective discussion with family members and medical providers. It was not unusual to observe five or six family members taking part in a treatment discussion. On the other hand, the LABC clinical trial (and its study sponsor) required a written consent document signed by the individual patient. The surgeon explained the difficulty in trying to reconcile these two methods:

There's typically a lot of extra information in the written consent form. It's not fair to expect a patient to understand all that information. They take the consent form and bring it home, but I don't think they read it. Their consent is really based on how comfortable they feel with us after one or two visits. Not on the form. There is a concern here that is very different from what you have in the West. Here the family is all-powerful. How to break the news and when? You have to take the family into all confidence with all decisions. Especially if the prognosis is bad, the family requests that the information be put in another way [conveyed in the least negative way possible].

We asked whether he felt that consent processes are for the family or for the patient? “I don't think it's for the patient. It's more for the family. Decisions are made by the family as a group. Or at least one or two members of the family. But usually, it's the father or the son or the sister who makes the decision. Then the patient signs. But this is only after the family gives the green signal.”

To our colleagues in Cochin, the wording of the consent was not the issue. The form was too long (15 pages). They guessed that patients would not read it (they were correct). Echoing investigators in Mexico and South Africa, investigators in India said that families would want to weigh the doctor's opinion of the trial in combination with their own. This was not because of literacy issues, although literacy was not unimportant, but because of expectations regarding communication. Furthermore, in cases where the patient was elderly or terminally ill, family members would sometimes opt to not tell the patient of her diagnosis, and this was generally considered acceptable by local physicians.

Our findings revealed that an expectation of individual consent was incommensurate with local expectations of collective decision-making; and a process that ended with a signature rather than a family agreement was not preferred. According to most of the providers we interviewed in India, Mexico, and South Africa, the consent form should be approached as a result and not the starting point for treatment conversation. In the end, because our colleagues in Cochin were not supported by the same study sponsor as the other sites, they were not beholden to the same IRB harmonization. Swiftly, they drew up a protocol that aligned with local convention: a short consent form that physicians could use as a reference point during consultations, but not expect patients to read.

#### Tangled in red tape: Divergent procedures and interests

As the trial was about to open in New York, we were on the phone with the collaborating surgeon in Mexico when he told us that he had some “very discouraging news.” The IRB in Mexico had decided that the transnational LABC center protocol (which had been revised at least four times to respond to specifications from national and local IRBs including Mexico) was so different from the original protocol that it could no longer be reviewed even as an amended protocol. The latest version would need to be resubmitted as an entirely new study. “This is really unfortunate,” explained our colleague, “because this means that the protocol goes to the back of the queue.” When we asked him, “so how long is the queue?” He replied, “I expect that it will take at least another 2 or 3 months to reach the point where the committee will look at it again.”

In the end, it took a year and a half to receive approval from the IRB in Mexico, and another 6 months for the study sponsor to give their green light. This was then followed by a period of at least 1 year during which the study PIs in New York would phone the colleague in Mexico City once a month to ask if there had been any progress with the study (namely, patient enrollment, tissue collection). Each time, the answer was “no, but I expect this will change.”

It appeared, at the outset, that institutional barriers were the cause of the delay. Typically, a high-volume clinic like the one in Mexico saw more than 150 breast cases per week, 10–15% of which might be confirmed as new diagnoses. This level of caseload gave physicians little time to speak with patients about their eligibility for a trial. In this case, however, it became clear that the lead physician on the study was not interested in the research. His initial assurance, in interviews, that he could do the job alone (independent of a multidisciplinary team) was probably a red flag. Coordinating the LABC study required the participation of nurses, data managers, social workers, and a raft of colleagues in surgery, medical oncology, radiotherapy, and pathology.

Fortunately, there was another researcher at the same center interested in taking the lead on the study. Right at that moment, however, a different set of issues emerged: a new administration had taken over the hospital, and they were not as interested in research. Their position was that medical practice and medical research should be carried out apart, and this created an epistemological flashpoint of the sort defined above. It was at that point, unfortunately, that the clinical collaboration fizzled. What did endure however was an interest in the investigation of structural problems impacting the high rates of advanced stage disease found at this particular institution.

#### Distributing for a common good: The ethics of sharing data and biological samples

The delays in regulatory approval that our collaborators experienced in Mexico had little to do with logistical or ethical dimensions of experimental research. During the 2 years of discussions leading up to the point that our collaborators received study approval, the national IRB in Mexico was more interested in the provenance of R&D components of the trial and made several rounds of requests for additional information regarding what biological (blood and tissue) samples would be collected, by whom, and in which laboratories. According to our colleagues in Mexico, the IRB wanted to know what opportunities for molecular and genetic training and technology transfer would be present if they signed onto the trial. In fact, our collaborator in Mexico himself strongly shared this interest and worked closely with us to cultivate research internships and international study exchanges for medical students and fellows between Mexico and the US.

The issue of tissue collection, shipment, and sharing also figured significantly in India. When asked whether the local IRB had raised concerns about the international dimension of the study, the PI in India responded: “Personally I don't feel strongly about that. But the government has been very concerned, especially with genetic therapies and engineering. Government agencies generally oppose sending any biological material abroad.” Due to anticipated roadblocks with the Indian Council of Medical Research (ICMR), tissue shipment was not attempted. In the end, the PIs in New York and India suspended the tissue sharing component of the collaboration. While the clinical trial and sociocultural studies proceeded, the possibility of biological material sharing remained firmly under the authority of the ICMR, an agency whose commitment to the national promotion of science made it reluctant to participate in collaborations where lab studies take place overseas. From their point of view, participation in studies where translational science or R&D takes place abroad prolonged the position of India as a site of resource extraction rather than innovation.

From an anthropological standpoint, the “sharing” of data or biological material was not neutral but fraught with legacies of extractivist science. This has been true as much for medicine as for anthropology; for example, ethnographic collecting expeditions deployed in colonial India sought to make anthropology worldly while producing Victorian-age science as authoritatively British (Breckenridge, 1989). The politics of data sharing raised questions about how information itself was interpreted. What was perceived as intellectual property in one cultural context was not always perceived the same way across sites; and the norms by which data sharing was practiced varied across borders. In regard to language and meaning, terms such as “non-proprietary” and “academic collaboration” were controversial and not easily agreed upon. Despite growing international acceptance of a general norm of data sharing across sites in the same academic program, there remained multiple and incompatible definitions of the term (For additional discussion of issues related to the collection and sharing of data sets across multiple sites or users, see, for example, Manderson et al., 2001 and Nygaard et al., 2007).

A better understanding of the multiple interpretations of “sharing” and its analogs (transfer, storage, extraction, translation, etc.) and application in different locations and contexts was critical to the facilitation of cooperation in this transnational study. Likewise, a clearer articulation of “common good” (rather than simply obligations and rights), especially in the area of data sharing, was greatly needed. Collaborating laboratories and scientists needed to see evidence (e.g., contractual plans rather than simply “good faith”) that the data they shared would be used for a global common good rather than just to increase the profits of Western medical institutions or biotech companies. Supporters of cross-site data sharing have argued that increased sharing enables researchers to better detect and respond to health threats of global significance such as COVID-19, SARS, and H1N1. Whether increased opportunities for data sharing translate into more robust systems of public health, however, is not clear and warrants greater discussion in the global cancer health community.

## Discussion

In the early days of the clinical trial, it was evident that our qualitative ethnographic approach would be valuable as a means toward more than sociocultural description of prolonged routes to breast cancer diagnosis and treatment. What if we could also tailor our approach toward a study of local system and public health dimensions that had already affected (or could affect) people's experiences of diagnosis, treatment, and post-treatment care. For example, specimen collection (tumor, blood) was an essential part of the clinical trial science, but it involved huge challenges. While some studies describe such challenges and how to address them (Ellerin et al., 2005), most trial reporting leaves them out, to the detriment of efforts to replicate similar procedures in other settings.

Our findings revealed two critical companion uses of ethnography in transnational cancer research. First, the value of ethnography as a mode of *evaluative listening*: appraising the perspectives of diverse patients and clinicians regarding prolonged routes to LABC diagnosis, treatment, and clinical trial decision making. Second, we show the value of ethnography as a *compass for navigating* among discrepant research styles, IRB protocols, and institutional norms and practices. At the same time, there are benefits and limits involved in each use of RER to be reconciled (or at the very least anticipated in a study of this scale). On the one hand, ethnographic interviews carried out before and during the clinical trial enabled more regular contact, social rapport, and communication among investigators and research sites. On the other hand, the logistics of doing the trial placed limits on the extent and duration of inductive, immersive inquiry characteristic of traditional fieldwork.

However, as the trial moved from the startup period where face-to-face check-ins, discussions, and troubleshooting were crucial, digital ethnographic interactions *via* video chat, text, and email added more contact and communication among investigators and the deepening of findings and analyses. Beyond what it sped up with regard to data collection and the potential application of results, it created what literary theorist Mikhail Bakhtin calls a *chronotope*, a process that spans space and time boundaries in a manner coming close to simultaneity (Bakhtin, 1981). This enabled a “just in time” dialogue about institutional politics and needs, in much the same way adverse events are reported in group trials and assessed by investigators across sites. This convergence among investigators otherwise separated by thousands of kilometers was particularly crucial when swift access to treatment was at stake. As air travel was already expensive, environmentally unfriendly, and time consuming to maintain the RER beyond only one or two initial site visits, we increasingly relied on hybrid ethnographic methods: an augmentation of video-chat, phone, text, and email carried out with, and built upon the initial connections established in, the in-person fieldwork. This style had its limits; but it did allow us to materially improve the ways in which the medical research proceeded.

In this way, the RER was chronotopic in its potential to bring disparate spaces, time differences, and diverse agendas together. At the same time, the *anticipatory* work of pre-trial ethnographic research was critical to understanding researcher differences, styles, and institutional practices and resources. The selection of sites and lead investigators was as critical to the success of the study as the study outcomes. No matter how well designed the study was for internal and external validity, no matter how valid the indicators, no matter how reliable the measurement tools, if the study was not (or could not be) implemented according to its design, the findings would not be reliable. In other words, if solid structures for communication and collaboration were not in place early during the development of multi-site research programs, nothing else would have been sustainable. At the same time, we needed greater awareness of the roles and contributions of various departments and agencies within the sites *before* the launch of the study. Early conversations should have included asking each collaborating department as well as each local PI what role they sought to play.

The difficulties we outline here took place long before the outbreak of the COVID-19 pandemic. The suddenness and speed of the pandemic's global spread intensified barriers to cancer treatment and clinical trials worldwide. Hospital closures and appointment cancellations led to a short-term drop in diagnoses, even as an uptick in more advanced diagnoses and mortality is now evident (Zhao et al., 2022). In light of the digital modalities accompanying COVID distancing protocols and quarantine, RER and multimodal approaches may shed light on hidden or unappreciated routes to diagnostic imaging or clinical care, while helping to promote ongoing communication among researchers during the course of a clinical trial, including when it has to shift most of its operations online. Just as traditional fieldwork is based on inductive science and uncertain results, rapid ethnographic research and multimodal ethnographic research models do not come with easy-to-follow directions or guaranteed benefits. Case analyses of the sort presented in this special issue of *Frontiers Medical Sociology* are therefore crucial for the sorts of relatable, if not replicable, guidance they may offer.

### The value of rapid ethnography in transnational cancer research

Much can be done to avoid problems that threaten success, and much can be learned from projects that do not unfold exactly as one expects. If there was one lesson that resounded above all others, it was that building a collaborative, connected team is essential and that the work involved in assembling a team can be as vital, prickly, and, in many ways, rewarding as piloting the research itself. Below, we summarize some potential benefits of RER in transnational cancer research.

#### Create curiosity

This may seem obvious but expectations regarding the value of research can differ dramatically among researchers. Aim for discussions early on with each investigator about why they are drawn to take part in the project. Expectations about discovery may not be shared. Brief life history interviews can be a great way to capture the interest and collaboration of multiple people, agendas, or institutional partners (Life history is an ethnographic method of exploring one person's lived experiences and how those shape the sorts of ways they see and live in the world).

#### Cultivate collaborators

Researchers do not tend to spontaneously start collaborating on their own. Because of its participatory, team-based approach, RER can promote “cooperation between experts and “non-experts” in problem solving” (Sangaramoorthy and Kroeger, 2020). RER can be a useful way to start with the assumption that perspectives and goals *will* be different. As counterintuitive as it may seem, expecting difference rather than agreement may result in a longer lasting collaboration.

#### Build a checklist

RER can enable researchers to identify local needs early on. Is the infrastructure sufficient to carry out the study protocol? Is each site equipped and prepared for the work? Does each site have ongoing capacity for collecting and tracking data? Are there site-specific IRB considerations, e.g., cultural expectations of informed consent, to consider early on?

#### Prioritize people

RER is a great way to make visible local structural issues that impact high burdens of diagnostic and treatment delay and then direct those findings into programs and policies that prioritize care for marginalized and vulnerable populations (Sangaramoorthy and Kroeger, 2020).

#### Promote public science

Rapid ethnography and digital ethnography can be used synergistically to create new forms of digital engagement, data sharing, and public science. This is potentially vital in situations where a healthcare problem is emerging or rapidly changing (Johnson and Vindrola-Padros, 2017; Vindrola-Padros et al., 2020).

### Future directions in global health

Efforts to create a team and to harmonize our approach gave us insights into bigger issues of public health. The reason for our study was to add to the knowledge needed to reduce deaths from breast cancer in regions where this burden remains especially high. However, the more we tried to identify and seek individuals within a community to be part of our study, the more deeply we entered into the community and their healthcare system. We found that delivery systems often lack preventive screening, or even rudimentary public health interventions. Referral from primary to specialty care (primary and secondary prevention) are defined differently and approached differently in different settings, as is the use of hospital-based medicine for anything other than acute care. The prevailing perception in many communities is that clinical treatment centers are bureaucratic and detached from social and family comforts. One makes use of these only during late stages of disease and only for urgent, acute interventions rather than for preventive (or even curative) care. In other words, the process of developing our research study gave us the impression that the lack of system capacity necessary for early detection and treatment of breast cancer plays an important role in the burden of advanced cancers globally and that social and community understanding are part of this gap.

As cancer research practice becomes ever more global, with similar shifts observable in public health and policy, we are likely to see the continuation of a trend whereby the borders that separate industry, academia, and advocacy become more porous. Just as HIV and AIDS activists established a new form of public engagement with clinical research over the past decades, cancer activist organizations have followed suit. This will change both the kind of research being conducted and the ethical and social terms used to ask people to take part in clinical cancer studies. Screening and early detection initiatives will succeed only when they achieve an alliance of organizations (governmental, legal, medical, educational) and only when they effectively address health service delivery factors such as availability, accessibility, and coordination between public health and medical services.

With this discussion, we have sought to show that there is a need for intensive, rapid ethnographic contact between countries, investigators, research participants, and advocates, and this contact should be in person and digital. Such approaches ensure findings can be adequately considered by diverse players (inside and outside an organization) and delivered to publics in an affordable way (Vindrola-Padros et al., 2020). In a majority of studies about cross-site cancer research, the emphasis has been on how to solve problems conceived in technical and legal terms (e.g., language to be tweaked in a consent form, data to be moved across borders). But lodged among the logistics of funding and ethics approvals is a world of epistemological differences; variations not only in what knowledge is to be communicated, but in how knowledge is approached in the first place. A more thorough evaluation of primary-to-specialty referral networks is needed to develop interventions aimed at reducing time to diagnosis, including improved training in early detection of smaller breast lesions and effective triage to diagnosis. In countries and medically underserved settings (including major public hospitals in the US) where advanced breast cancer accounts for a high burden of cases, such interventions may enable significant improvements in breast cancer related morbidity and mortality, while reducing the associated high costs for people diagnosed with this disease as well as the medical systems that care for them.

## Data availability statement

The raw data supporting the conclusions of this article will be made available by the authors, without undue reservation.

## Ethics statement

The studies involving human participants were reviewed and approved by NYU School of Medicine IRB (New York, NY), Bellevue Hospital Center IRB (New York, NY), Tygerberg Hospital IRB (Cape Town, South Africa), Instituto Mexicano del Seguro Social IRB (Mexico City, Mexico), Amrita Institute of Medical Sciences (Cochin, India), and US Congressionally Directed Medical Research Program/Department of Defense IRB (Fort Detrick, Maryland). Written informed consent for participation was not required for this study in accordance with the national legislation and the institutional requirements. Written informed consent was obtained from the individual(s) for the publication of any potentially identifiable images or data included in this article.

## Author contributions

KB designed and carried out the study, performed analysis, and wrote the manuscript.

## Funding

This work was supported by the US Congressionally Directed Medical Research Program under DAMD17-04-1-0905 and the Breast Cancer Research Foundation.

## Conflict of interest

The author declares that the research was conducted in the absence of any commercial or financial relationships that could be construed as a potential conflict of interest.

## Publisher's note

All claims expressed in this article are solely those of the authors and do not necessarily represent those of their affiliated organizations, or those of the publisher, the editors and the reviewers. Any product that may be evaluated in this article, or claim that may be made by its manufacturer, is not guaranteed or endorsed by the publisher.

## References

[B1] Adams S. Chakravarthy A. B. Donach M. Spicer D. Lymberis S. Singh B.. (2010). Preoperative concurrent paclitaxel-radiation in locally advanced breast cancer: pathologic response correlates with five-year overall survival. Breast Cancer Res. Treat. 124, 723–732. 10.1007/s10549-010-1181-8 20878462 PMC3655407

[B2] American Cancer Society (ACS). (2022). Cancer Facts and Figures 2022. Atlanta: American Cancer Society. Available online at: https://www.cancer.org/research/cancer-facts-statistics/all-cancer-facts-figures/cancer-facts-figures-2022.html (accessed June 29, 2022).

[B3] American Society of Clinical Oncology (ASCO). (2022). Breast Cancer: Statistics. Available online at: https://www.cancer.net/cancer-types/breast-cancer/statistics (accessed July 9, 2022).

[B4] Arslan A. A. Silvera D. Arju R. Giashuddin S. Belitskaya-Levy I. Formenti S. C.. (2012). Atypical ezrin localization as a marker of locally advanced breast cancer. Breast Cancer Res. Treat. 134, 981–988. 10.1007/s10549-012-2017-5 22415480

[B5] Bakhtin M. (1981). “Forms of time and of the chronotope in the novel*,”* in *The Dialogic Imagination. Ed. Michael Holquist. Trans. Caryl Emerson and Michael Holquist*. Austin: U of Texas Press.

[B6] Balogun O. D. Formenti S. C. (2015). Locally advanced breast cancer–strategies for developing nations. Front. Oncol. 5, 89. 10.3389/fonc.2015.00089 25964882 PMC4410621

[B7] Banerjee D. (2020). Enduring Cancer: Life, Death, and Diagnosis in Delhi. Durham, NC: Duke University Press.

[B8] Braunstein S. Karpisheva K. Pola C. Goldberg J. Hochman T. Yee H.. (2007). A hypoxia-controlled cap-dependent to cap-independent translation switch in breast cancer. Mol. Cell 28, 501–512. 10.1016/j.molcel.2007.10.019 17996713

[B9] Breckenridge C. A. (1989). The aesthetics and politics of colonial collecting: India at world fairs. Comp. Stud. Soc. Hist. 31, 195–216. 10.1017/S0010417500015796

[B10] Bright K. (2015). “Love in the time of cancer: kinship, memory, migration and other logics of care in Kerala, India,” in Anthropologies of Cancer in Transnational Worlds. Abingdon; Oxfordshire, UK; New York, NY: Routledge. 149–169.

[B11] Bright K. Barghash M. Donach M. de la Barrera M. G. Schneider R. J. Formenti S. C. (2011). “The role of health system factors in delaying final diagnosis and treatment of breast cancer in Mexico City, Mexico. Breast 20, S54–S59. 10.1016/j.breast.2011.02.012 21371885

[B12] Bright K. Schneider R. J. Goldberg J. D. Vijaykumar D. K. Apffelstaedt J. de la Barrera M. G.. (2008). “Structural and cultural barriers to clinical diagnosis of locally advanced breast cancer (LABC) in a multiethnic cohort: findings from Mexico, South Africa, India, and the United States,” in Proceedings of the United States Congressionally Directed Medical Research Program and Department of Defense Breast Cancer Research Program. Fort Detrick„ MD: United States Congressionally Directed Medical Research Program.

[B13] Burke N. J. (2014). ”Rethinking the therapeutic misconception: social justice, patient advocacy, and cancer clinical trial recruitment in the US safety net. BMC Med. Ethics 15, 1–7. 10.1186/1472-6939-15-68 25240404 PMC4177718

[B14] Caduff C. Skelton M. Banerjee D. Djordjevic D. Mika M. Mueller L.. (2018). Analysis of social science research into cancer care in low-and middle-income countries: improving global cancer control through greater interdisciplinary research. J. Glob. Oncol. 4, 1–9. 10.1200/JGO.18.00045 30084699 PMC6223507

[B15] Caplan L. (2014). Delay in breast cancer: implications for stage at diagnosis and survival. Front. Public Health 2, 87. 10.3389/fpubh.2014.00087 25121080 PMC4114209

[B16] Connolly E. Braunstein S. Formenti S. Schneider R. J. (2006). Hypoxia inhibits protein synthesis through a 4E-BP1 and elongation factor 2 kinase pathway controlled by mTOR and uncoupled in breast cancer cells. Mol. Cell. Biol. 26, 3955–3965. 10.1128/MCB.26.10.3955-3965.2006 16648488 PMC1489005

[B17] Eaton T. Bright K Zeng X. Thompson H. S. (2017). Chinese American immigrant breast cancer survivors and their experiences with post-treatment care. J. Health Dispar. Res. Pract. 10, 181–203. Available online at: https://digitalscholarship.unlv.edu/jhdrp/vol10/iss1/11

[B18] Ellerin B. E. Schneider R. J. Stern A. Toniolo P. G. Formenti S. C. (2005). Ethical, legal, and social issues related to genomics and cancer research: the impending crisis. J. Am. Coll. Radiol. 2, 919–926. 10.1016/j.jacr.2005.03.016 17411966

[B19] Formenti S. C. Volm M. Skinner K. A. Spicer D. Cohen D. Perez E.. (2003). Preoperative twice-weekly paclitaxel with concurrent radiation therapy followed by surgery and postoperative doxorubicin-based chemotherapy in locally advanced breast cancer: a phase I/II trial. J. Clin. Oncol. 21, 864–870. 10.1200/JCO.2003.06.132 12610186

[B20] Hendrick R. E. Monticciolo D. L. Biggs K. W. Malak S. F. (2021). Age distributions of breast cancer diagnosis and mortality by race and ethnicity in US women. Cancer 127, 4384–4392. 10.1002/cncr.33846 34427920

[B21] Johnson G. A. Vindrola-Padros C. (2017). Rapid qualitative research methods during complex health emergencies: a systematic review of the literature. Soc. Sci. Med. 189, 63–75. 10.1016/j.socscimed.2017.07.029 28787628

[B22] Joseph G. Dohan D. (2012). Recruitment practices and the politics of inclusion in cancer clinical trials. Med. Anthropol. Q. 26, 338–360. 10.1111/j.1548-1387.2012.01222.x 23259347

[B23] Livingston J. (2012). Improvising Medicine: An African Oncology Ward in an Emerging Epidemic. Duke University Press.24962205

[B24] Mabry P. L. Pronk N. P. Amos C. I. Witte J. S. Wedlock P. T. Bartsch S. M.. (2022). Cancer systems epidemiology: Overcoming misconceptions and integrating systems approaches into cancer research. PLoS Med. 19, e1004027. 10.1371/journal.pmed.1004027 35714096 PMC9205504

[B25] Manderson L. Kelaher M. Woelz-Stirling N. (2001). Developing qualitative databases for multiple users. Qual. Health Res. 11, 149–160. 10.1177/104973201129119019 11221112

[B26] Nygaard P. Bright K. Saltz R. McGaffigan R. (2007). Archival data: collection and use in community alcohol projects. Subst. Use Misuse 42, 1945–1953. 10.1080/10826080701532965 18075919

[B27] Palinkas L. A. Whiteside L. Nehra D. Engstrom A. Taylor M. Moloney K.. (2020). Rapid ethnographic assessment of the COVID-19 pandemic April 2020 ‘surge'and its impact on service delivery in an Acute Care Medical Emergency Department and Trauma Center. BMJ Open 10, e041772. 10.1136/bmjopen-2020-041772 33082198 PMC7577068

[B28] Petryna A. (2009). When Experiments Travel: Clinical Trials and the Global Search for Human Subjects. Princeton, NJ: Princeton University Press. http://press.princeton.edu/chapters/i8916.html

[B29] Rapport F. Smith J. O'Brien T. A. Tyrrell V. A. Mould E. V. A. Long J. C.. (2020). Development of an implementation and evaluation strategy for the Australian ‘Zero Childhood Cancer'(Zero) Program: a study protocol. BMJ Open 10, e034522. 10.1136/bmjopen-2019-034522 32580982 PMC7312332

[B30] Sangaramoorthy T. Kroeger K. A. (2020). Rapid Ethnographic Assessments: A Practical Approach and Toolkit for Collaborative Community Research. Abingdon; Oxfordshire, UK; New York, NY: Routledge.

[B31] Unger-Saldaña K. (2014). Challenges to the early diagnosis and treatment of breast cancer in developing countries. World J. Clin. Oncol. 5, 465. 10.5306/wjco.v5.i3.465 25114860 PMC4127616

[B32] Unger-Saldaña K. Fitch-Picos K. Villarreal-Garza C. (2019). Breast cancer diagnostic delays among young Mexican women are associated with a lack of suspicion by health care providers at first presentation. J. Glob. Oncol. 5, 1–12. 10.1200/JGO.19.00093 31335236 PMC6690634

[B33] Villarreal-Garza C. Lopez-Martinez E. A. Muñoz-Lozano J. F. Unger-Saldaña K. (2019). Locally advanced breast cancer in young women in Latin America. Ecancermedicalscience 13, 894. 10.3332/ecancer.2019.894 30792811 PMC6372300

[B34] Villarreal-Garza C. Mohar A. Bargallo-Rocha J. E. Lasa-Gonsebatt F. Reynoso-Noverón N. Matus-Santos J.. (2017). Molecular subtypes and prognosis in young Mexican women with breast cancer. Clin. Breast Cancer 17, e95–e102. 10.1016/j.clbc.2016.11.007 28065398

[B35] Vindrola-Padros C. Chisnall G. Cooper S. Dowrick A. Djellouli N. Symmons S. M.. (2020). Carrying out rapid qualitative research during a pandemic: emerging lessons from COVID-19. Qual. Health Res. 30, 2192–2204. 10.1177/1049732320951526 32865149 PMC7649912

[B36] Vindrola-Padros C. Vindrola-Padros B. (2018). Quick and dirty? A systematic review of the use of rapid ethnographies in healthcare organisation and delivery. BMJ Qual. Saf. 27, 321–330. 10.1136/bmjqs-2017-007226 29263139

[B37] World Health Organization (WHO). (2022). Cancer. Available online at: https://www.who.int/news-room/fact-sheets/detail/cancer (accessed June 29, 2022).

[B38] Yip C. H. Anderson B. O. Bright K. Caleffi M. Cardoso F. Elzawawy A. M.. (2011). Breast cancer management in middle-resource countries (MRCs): consensus statement from the Breast Health Global Initiative. Breast 20, S12–S19. 10.1016/j.breast.2011.02.015 21388811

[B39] Zhao J. Han X. Miller K. Zheng Z. Nogueira L. M. Islami F.. (2022). Changes in cancer-related mortality during the COVID-19 pandemic in the United States. J. Clin. Oncol. 40, (suppl 16), 6581. 10.1200/JCO.2022.40.16_suppl.658133683506

